# Fully Digital Workflow for Planning Static Guided Implant Surgery: A Prospective Accuracy Study

**DOI:** 10.3390/jcm9040980

**Published:** 2020-04-01

**Authors:** Chia-Cheng Lin, Ching-Zong Wu, Mao-Suan Huang, Chiung-Fang Huang, Hsin-Chung Cheng, Dayen Peter Wang

**Affiliations:** 1 School of Dentistry, College of Oral Medicine, Taipei Medical University, Taipei 110, Taiwan; 2 Department of Dentistry, Shin Kong Wu Ho-Su Memorial Hospital, Taipei 111, Taiwan; 3 Department of Dentistry, Taipei Medical University Hospital, Taipei 110, Taiwan; 4 Department of Dentistry, Taipei Medical University-Shuang Ho Hospital, New Taipei City 235, Taiwan; 5 School of Oral Hygiene, College of Oral Medicine, Taipei Medical University, Taipei 110, Taiwan; 6 School of Dental Technology, College of Oral Medicine, Taipei Medical University, Taipei 110, Taiwan

**Keywords:** digital workflow, guided implant surgery, stereolithographic surgical guide, accuracy, CAD/CAM, clinical research

## Abstract

The accuracy of static guided implant surgery (sGIS) using conventional planning workflow has been extensively examined; however, more information is required to justify the application of fully digital planning protocol. The purpose of this study was to investigate the clinical accuracy of sGIS with a fully digital planning workflow. Twenty-one partially edentulous patients were enrolled in this prospective study. Cone-beam computed tomography (CBCT) and intraoral scans were taken and superimposed by matching the dental surface images directly (surface registration protocol) or by matching fiducial markers on a stereolithographic (SLA) radiographic template fabricated from the digital data of the intraoral scan (fiducial marker registration protocol). Virtual implant treatment plans were then determined, and tooth-supported SLA surgical guides were fabricated according to the plans. Twenty-six implant surgeries were performed via the surgical guide by one surgeon. Pre- and post-operative CBCT images were superimposed, and the positional and angular deviations between placed and planned implants were measured with metrology software. A total of 43 fully guided implants were placed, in which 25 implants were planned with the surface registration protocol. Implants planned based on the surface registration protocol had a larger mean angular deviation than the fiducial marker registration protocol. No significant differences were found for any deviations of the examined variables. Within the limits of this study, we concluded that the clinical accuracy of the sGIS planned with a fully digital workflow was consistent with the conventional workflow for partially edentulous patients.

## 1. Introduction

Implant dentistry has shifted from a “surgically driven approach” to a “prosthetically driven approach”, because appropriate implant positioning is crucial to achieving long-term functional and esthetic success [1]. In the meantime, computer-guided implant surgery has been developed for over two decades. This digital technology is now commonly recommended because it can reduce inaccuracy in terms of implant positioning [2]. To date, two types of guided implant surgery systems have been developed: static (template-based) and dynamic navigation. The static approach (static guided implant surgery, sGIS) refers to the use of a surgical guide (drilling template) in implant surgery. The surgical guide can confine (guide) the direction and depth of the implant bed preparation and implant placement so that a virtually planned implant position can be transferred to the implant site. Currently, the static system is more popular, because dynamic navigation needs additional expense and space for the equipment [3]. However, to achieve the appropriate implant position and angulation, a precise blueprint of the future restoration on the implant, that is, the digital planning itself, is an essential determinant of the correct implant positioning for computer-guided implant surgery.

The digital implant planning is virtually performed on 3D images of the jawbone acquired from computed tomography (CT) or cone-beam computed tomography (CBCT). The latter is nowadays more popular because of the relatively lower radiation dose and cost [1]. Using 3D planning software, the ideal implant size and location in the alveolar bone was designed according to the position and contour of the desired restoration. Conventionally, in order to take into account the future restoration in the implant site during implant planning, a special scan prosthesis with radiopaque teeth or markers was necessary [4]. The scan prosthesis was fabricated in the laboratory on a dental model according to the diagnostic wax-up of the patient. The patient wore the scan prosthesis to take a CBCT scan so that the image of the planned prosthetic setup could be incorporated into the anatomic image of the jawbone, then the ideal implant position could be planned using 3D planning software. The disadvantages of the scan prosthesis are that extra time and cost needed for the laboratory fabrication procedure. Human errors during the laboratory procedure could happen. Moreover, an incorrect intraoral seating of the scan prosthesis during the CBCT scan would lead to a wrong planning position of the implant [5].

With the evolution of digital technology and apparatus, a new “image-fusion” technique was introduced and dramatically reduced the time and cost of the planning procedure [6,7,8]. For partially edentulous patients, a CBCT scan image can be matched with an optical scan image of the dental model, directly by registration (alignment) of the dental surface images via the computer algorithm. Digital wax-up of the missing teeth can also be performed so that the implant planning can be done in the same software. Errors from the fabrication and scanning of a radiographic scan prosthesis can be avoided. Excellent in vitro accuracy was reported for this protocol [6]. However, conventional impression procedure and plaster models are necessary for this protocol, which means additional time and cost are still needed.

About the same time, intraoral scanners were implemented in guided implant surgery [9,10,11]. In addition, a direct match of intraoral scan and CBCT scan was described [12,13], and a comparable in vitro accuracy was reported between CBCT registration with an intraoral scan and with a laboratory scan [14]. The accuracy (precision and trueness) of digital impression by desktop scanners and intraoral scanners has been examined to be excellent [15]. By combing the technique of intraoral scanning and the algorithm of dental surface registration, a fully digital workflow for planning the sGIS can be established. The resulting digital workflow can be more time efficient, and human errors can be decreased owing to the simplified procedures.

In the literature, the accuracy of sGIS using conventional planning workflow had been extensively examined. [2,16,17,18,19,20,21]. One in vivo study investigating the accuracy of the sGIS by a fully digital planning modality has been reported [22]. A comparable result to conventional guided implant surgery was observed. However, more information is required to justify the application of this fully digital planning protocol. The purpose of this study, therefore, was to investigate the clinical accuracy of the sGIS planned by a fully digital workflow.

## 2. Materials and Methods

### 2.1. Patient Enrolment

The study protocol was approved by the ethical committee of Shin Kong Wu Ho-Su Memorial Hospital, Taipei, Taiwan (20150708R). Thirty patients were recruited in the study period between January 1, 2016, and June 30, 2017, after having signed the informed consent. The inclusion criterion was partially edentulous patients with a healthy periodontium. The excluded patients included patients with major systemic diseases, the need for extensive bone grafting in planned implant site, pregnancy, under bisphosphonate treatment, and limited mouth-opening for executing the sGIS. The patients underwent a complete oral and radiographic examination before inclusion in this study, and a total of 21 patients were included after the screening.

### 2.2. Alignment of CBCT Scan and Intraoral Scan

All included patients had a CBCT scan of the entire jaw, using a large field-of-view CBCT scanner (3D eXam, KaVo Dental, Biberach, Germany) with a voxel size of 0.25 mm. Maxillary and mandibular digital impressions and the interocclusal registration were acquired using an intraoral scanner (TRIOS 3, 3Shape, Copenhagen, Denmark). For the superimposition of the CBCT scan and the intraoral scan, two protocols were adopted according to the patient’s dental conditions:

#### 2.2.1. Surface Registration Protocol

**Figure 1:**
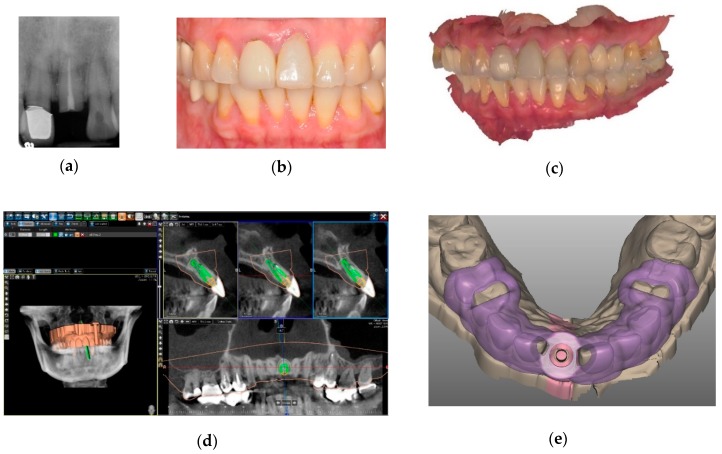
Surface registration protocol of implant planning. (**a**) A radiograph of a fractured left maxillary central incisor. (**b**) A temporary restoration for esthetics was fabricated on the root with fiber post and resin composite. (**c**) An image of maxillary and mandibular digital impressions after occlusal registration. (**d**) A cone-beam computed tomography (CBCT) image matched with an intraoral scan by dental surface registration algorithm, with ideal implant position planned (green). (**e**) A computer-aided design (CAD) image of the designed surgical guide.

The Digital Imaging and Communication in Medicine (DICOM) files of the CBCT scan and the Standard Tessellation Language (STL) files of the intraoral scan were imported into implant planning software (BenQ AB Guided Service, Ashdod, Israel) and were matched with the registration tool of the software by setting corresponding point sets on the dental surface images, as seen in Figure 1.

#### 2.2.2. Fiducial Marker Registration Protocol

**Figure 2:**
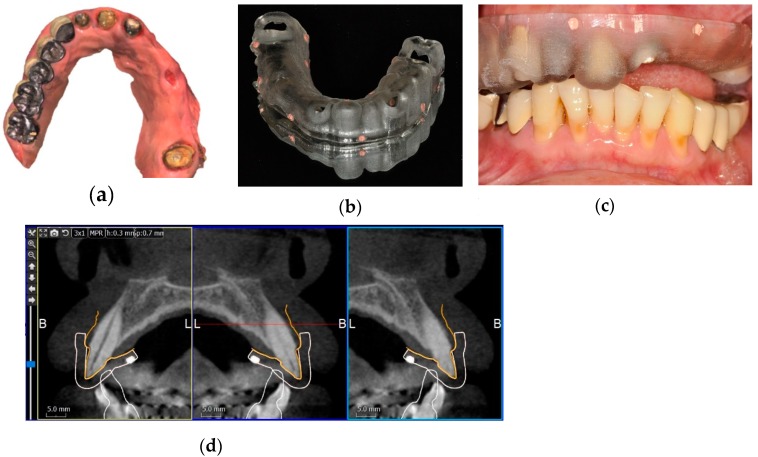
Fiducial marker protocol of implant planning. (**a**) An occlusal image of a maxillary intraoral scan. Five metal crowns on the right side and the inadequate number of residual teeth made the correct alignment of CBCT and intraoral scans by dental surface registration difficult. (**b**) Using the digital data of the intraoral scan, a stereolithographic (SLA) radiographic template with gutta-percha fiducial markers was fabricated. (**c**) The SLA radiographic template in the mouth ready for a CBCT scan. (**d**) A CBCT image aligned with the intraoral scan image (orange line) by matching the fiducial markers (white cubes) on the SLA radiographic template (white line).

For some patients, due to the extensive streaking artifact of metal crowns in the CBCT image or an inadequate number of remaining teeth, the correct alignment of the CBCT and intraoral scan images was challenging to achieve by dental surface registration. In such a case, a protocol similar to the conventional procedure was adopted. A stereolithographic (SLA) radiographic template with radiopaque fiducial markers was designed and fabricated according to the digital data of the intraoral scan. Five to six fiducial markers were evenly distributed on each side (buccal and lingual) of the radiographic template. Their positions were away from the clinical crowns of the teeth to facilitate the correct mapping. The patient took a CBCT scan wearing the SLA radiographic template on the remaining teeth. In this way, the position of the radiographic template on the CBCT scan could be identified by the radiopaque fiducial markers. Because the digital image of the radiographic template can coincide well with the intraoral scan, the position of the intraoral scan image and its geometry can be attained through the radiographic template. This CBCT image, therefore, could be more correctly superimposed with the intraoral scan by matching the fiducial markers on the radiographic template, as seen in Figure 2.

### 2.3. Implant Planning and Surgical Guide Fabrication

**Figure 3:**
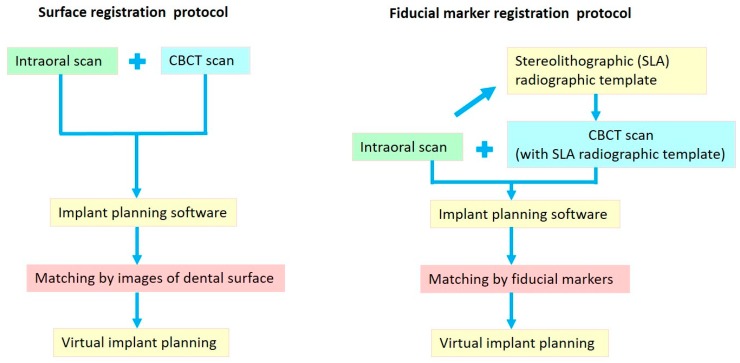
Workflow of digital implant planning.

The resulting alignment was visually examined by a technician to check the correctness and repeat the procedure if necessary. Once the alignment was verified, the surgeon could then perform a virtual implant treatment plan with the software. Considering the ideal prosthetic and anatomic conditions, an implant with proper diameter and length was virtually planned with the implant planning software. The digital implant treatment planning was then exported in STL format to computer-aided design (CAD) software (PlastyCAD, 3DIEMME Bioimaging Tecnologies, Figino Serenza, Italy), and the surgical guide was designed according to this planned implant position. The completed CAD files of the surgical guide were then uploaded to the computer-aided manufacturing (CAM) software (GrabCAD Print, Stratasys Ltd., Eden Prairie, MN, USA) of a 3D printer (Objet260 Connex, Stratasys Ltd.), and an SLA surgical guide (BenQ AB Guide, BenQ AB DentCare Corp., Taipei, Taiwan) was fabricated by a certificated manufacturer using the modeling resin material (MED610, Stratasys Ltd.) with the following settings: 2 mm of thickness and 0.08 mm of guide-to-teeth offset. Metal sleeves (4.5 mm in height and 5.0 mm in diameter) specific to the surgical guide system were inserted into the surgical guide. All the surgical guides were tooth-supported. Figure 3 illustrated the workflow of the digital implant planning.

### 2.4. Surgical Procedure

**Figure 4:**
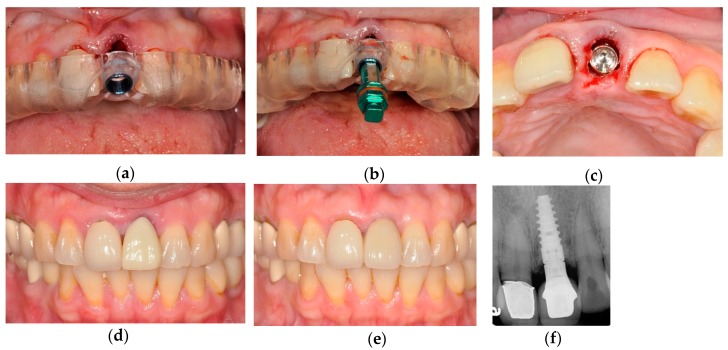
Clinical procedures of the patient in Figure 1. (**a**) An SLA surgical guide was verified for correct seating. (**b**) Fully guided implant placement with the fixture mount. (**c**) An intraoral view of placed implant. (**d**) Provisional restoration the next day after surgery. (**e**) Finished restoration after 6 months of healing. (**f**) Radiograph of the finished restoration after 2 years.

All the implant surgeries were performed by one experienced doctor (C.-C.L.). Before implant surgery, the SLA surgical guide was verified in the patient’s mouth for correct seating and stability. If adequate alveolar bone width (5 mm) and keratinized gingival tissue (4.5–5 mm) were available in the implant site, a flapless approach was performed after block or infiltration anesthesia application; otherwise, an open flap surgery was performed. All the guided surgeries were performed using the BenQ AB Guided Service System (BenQ AB DentCare Corp., Taipei, Taiwan). Drill keys with varying inner diameters and their corresponding drills were used sequentially. All drills had a physical stop on the top of the drill to allow depth control. Following the standard protocol of the surgical guide system, guided drilling procedures were executed, and implant fixtures were inserted through the surgical guide sleeve (fully guided insertion). Internal hex, bone level type implants (I5 Conical Implant, AB dental, Ashdod, Israel) with varying length (8–16 mm) and diameter (3.5–4.2 mm) were used for all patients. An example of clinical procedures is concisely presented in Figure 4.

### 2.5. Deviation Measurement

With the approval of the ethical committee and the informed consent of the patients, a post-operative CBCT scan was checked immediately after the implant placement. The pre- and post-operative CBCT images of the jaw were superimposed using the registration algorithm of the aforementioned implant planning software, so the virtually planned implant position could be compared with the placed implant position in the same data set.

**Figure 5:**
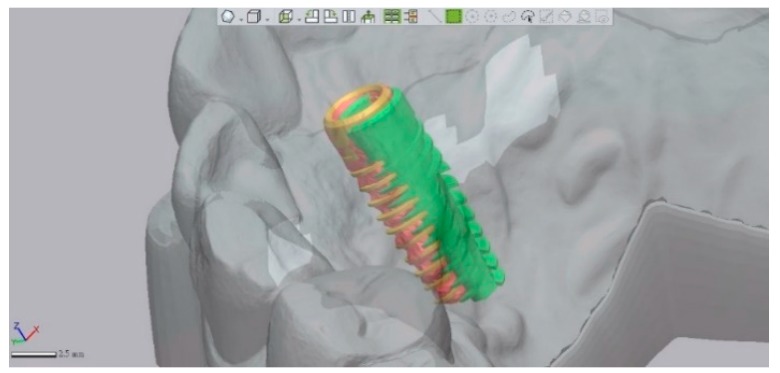
A CAD file (brown) of the implant replaced the ambiguous post-operative CBCT implant image (red) by using the best-fit algorithm, so that the deviation between the planned implant (green) could be more appropriately measured.

Because of the streaking effect of the metal artifacts, the implant image of the post-operative CBCT was ambiguous at this point, and the determination of the correct geometry could be challenging. Therefore, the data set was exported to a CAD software (Geomagic Design X, 3D Systems Inc., Rock Hill, SC, USA) in STL format. Using the best-fit algorithm of the software, the ambiguous post-operative implant image was then extracted and replaced by the CAD files corresponding to the placed implant. Hence, a virtual implant best fitted the geometry and the position of the post-operative implant image was attained, and the deviations between planned and placed implants could be measured more correctly, as seen in Figure 5.

**Figure 6:**
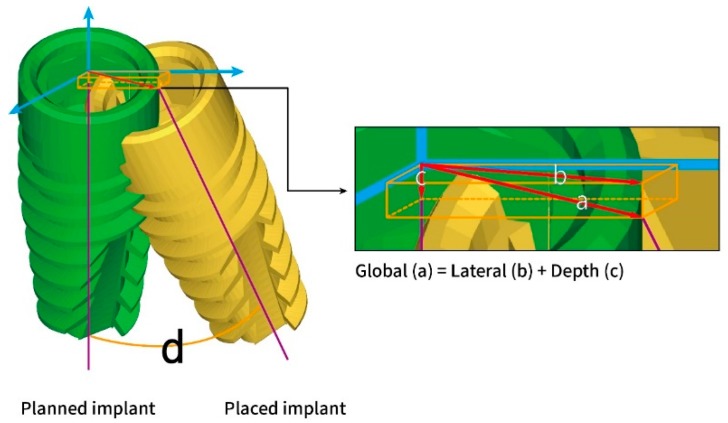
Definitions of the deviations between planned and placed implants at the implant platform: (**a**) global deviation, (**b**) lateral deviation, (**c**) depth deviation, and (**d**) angular deviation. The deviations at the implant apex were defined and measured by the same coordinate system (blue arrows).

**Figure 7:**
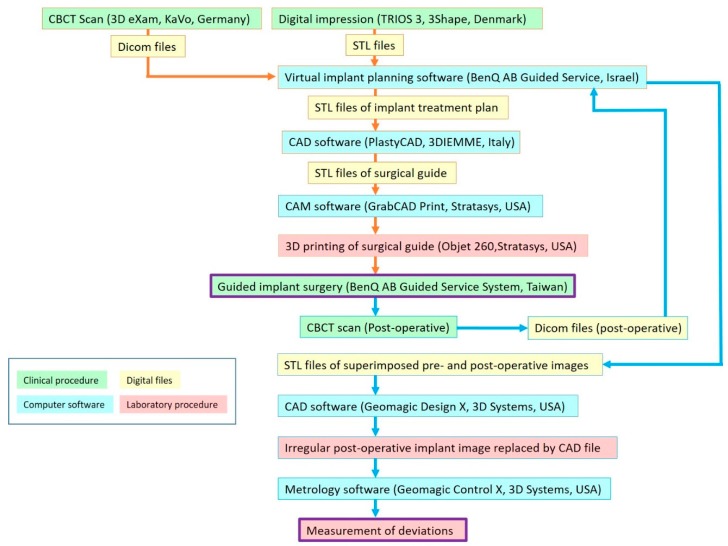
Summarized workflow of guided surgery (orange arrows) and deviation measurement (blue arrows).

The digital files were then exported to a metrology software (Geomagic Control X, 3D Systems Inc.). The planned and placed implant images could be determined with the software and their deviations could then be measured. The following positional and angular deviations of interest were defined and calculated: global deviation at the implant platform/apex, lateral deviation at the implant platform/apex, depth deviation, and angular deviation, as seen in Figure 6. The global deviation is the spatial distance between the center of the implant platform/apex of planned and placed implants. The lateral deviation is the directional component of the global deviation at the level of the planned implant platform/apex. The depth deviation is the distance of planned and placed implants on the axis of the planned implant. The angular deviation is the spatial angle between the planned and placed implant axis. A schematic diagram is shown in Figure 7 summarizing the workflow of the guided surgery and the deviation measurement.

### 2.6. Statistical Analysis

Sample size calculations were based on the data from a pilot study on dental model. According to the one-way ANOVA, an effect size of 0.816 was calculated from the means of three groups of the pilot data. Hence, a total sample size of 21 was obtained using the effect size of 0.8, 5% α-error, and 80% power. Because more than one implant would be expected to be placed in each patient, this sample size was considered sufficient to detect the intergroup differences.

The outcome variables of interest were the global deviation at the implant platform/apex, the lateral deviation at the implant platform/apex, the depth deviation, and the angular deviation. The predictor variables that could affect the accuracy of the guided implant surgery were defined and categorized as follows: the location of the implant site (incisor/canine, premolar, molar), the jaw position of the implant site (maxilla or mandible), the bone quality of the implant site (type II, III, IV), implant length (≤10 mm, 11.5 mm, ≥13 mm), implant diameter (3.5 mm, 3.75 mm, 4.2 mm), the support of the surgical guide (bilaterally tooth-supported or unilaterally tooth-supported in distal extension situation), and the surgical approach (open flap or flapless procedure). Mean value, standard deviation, and range were used to describe the quantitative data. Box plots were drawn to show the descriptive distribution of the deviation data. The two-sample t-test and the analysis of variance (ANOVA) test were used to examine the intergroup differences. Data analysis was carried out using STATA 14 statistics software (StataCorp, College Station, TX, USA). The significance level was set at *p* = 0.05.

## 3. Results

The 21 patients included (10 males and 11 females) had a mean age of 59.6 years (range: 37–83 years). The digital treatment plans of 15 patients were based on the surface registration protocol, whereas six patients were based on the fiducial marker protocol. Five patients had two implant surgeries in different regions, so a total of 26 sGIS were performed as planned. A total of 50 implants were installed. However, seven implants in five surgeries had to be inserted free-handed in the molar sites due to the limited mouth opening of the patient; hence, only 43 implants were inserted fully guided by the surgical guide. No complications of the surgical guide, such as the fracture of the guide or the detachment of the metal sleeves were observed during the surgery. Two implants in one patient were removed on account of pain after four weeks of healing, yet no signs of infection, such as swelling or abscess formation, were noticed for this patient. Besides this patient, all the other implants were successfully restored, including nine single tooth gaps and 16 edentulous ridges of over two missing teeth.

Only the deviations of fully guided implants were included for statistical analysis. For all 43 fully guided implants, the mean global deviations at implant platform/apex were 0.78 ± 0.39/1.28 ± 0.72 mm. The mean lateral deviations at implant platform/apex were 0.57 ± 0.33/1.14 ± 0.72 mm. The mean depth and angular deviations were 0.46 ± 0.36 mm and 4.30 ± 2.87º, respectively. Together with the data of 43 fully guided implants, Table 1 presents and compares the results between matching CBCT and intraoral scans by dental surface images (surface registration protocol) and by fiducial markers on the radiographic template (fiducial marker registration protocol). Implants planned based on the surface registration protocol showed significantly larger lateral deviation at the implant apex (1.34 ± 0.86 mm) than the fiducial marker registration protocol (0.87 ± 0.30 mm, *p* = 0.0167). Moreover, marginal significances were observed for the global deviation at the implant apex and the angular deviation (*p* = 0.0588 and *p* = 0.0526, respectively). Implants planned based on the surface registration protocol had a larger mean angular deviation than the fiducial marker registration protocol.

Table 2 shows the comparison of deviations between placed and planned implants for each of the examined variables of the patient, implant, surgical guide, and surgical technique. No significant differences of any positional or angular deviations were found for any of the variable groups.

**Figure 8:**
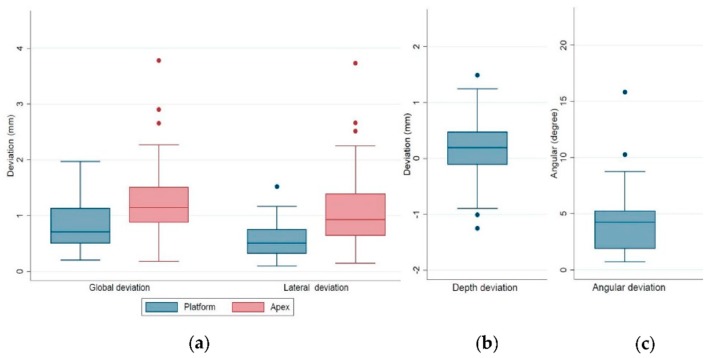
Box plots of the (**a**) global and lateral deviations at implant platform/apex, (**b**) depth deviation, and (**c**) angular deviation.

The distributions of the analyzed deviations are shown in detail in the box plots in Figure 8. In general, the deviations at implant apex were larger than at the implant platform: 75% of the values of the global/lateral deviation at the implant platform were below 1.13/0.75 mm (upper quartile, Figure 8a); 50% of the values of depth deviation were between +0.47 mm (upper quartile) and −0.11 mm (lower quartile, Figure 8b); and 75% of the values of the angular deviation were below 5.23º (upper quartile), with two outliers above 10º (Figure 8c).

**Figure 9:**
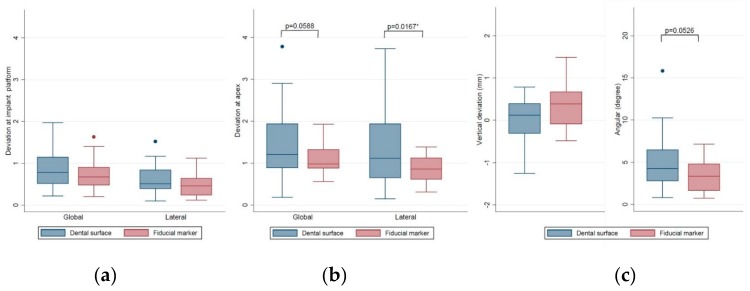
Box plots of the deviations of surface registration protocol (blue) and fiducial marker registration protocol (red). (**a**) The global and lateral deviations at the implant platform, (**b**) global and lateral deviations at the implant apex, and (**c**) the depth and angular deviations.

Figure 9 presents the distributions of deviations and the comparison between surface registration protocol and fiducial marker registration protocol. In general, implants planned with the surface registration protocol had larger values of global and lateral deviations at implant apex (Figure 9b), and angular deviations (Figure 9c, right) than implants planned with the fiducial marker protocol.

## 4. Discussion

The implementation of digital technology has influenced the dentistry at a great pace [23]. On account of the progress of CAD/CAM systems, the digital workflow is continually growing in dentistry and is considered smooth and time-efficient [24]. With the development of new-generation intraoral scanners, digital impression was reported to have advantages such as reducing patient discomfort, diminishing the possible deformation of impression materials, decreasing the potential cost, and increasing efficacy [25,26]. This has been applied in various fields of dentistry, including prosthodontics, orthodontics, implantology, and even tumor treatment [26,27]. Although the application of intraoral scanners could simplify the workflow and reduce the costs of computer-guided implant surgery [27], it has not been widely used in sGIS to date [26].

The accuracy of sGIS using conventional planning workflow has been widely investigated [2,16,17,18,19,20,21]. To our best knowledge, however, only one prospective study has examined the in vivo accuracy of sGIS based on a fully digital planning procedure. Skjerven et al. [22] examined the in vivo accuracy of 27 implants in 21 patients using a fully digital planning modality and SLA surgical guides. The mean lateral deviation at the implant platform/apex, depth deviation, and angular deviation were 1.05 mm/1.63 mm, +0.48 mm, and 3.85º, respectively. The authors concluded that the results of this simplified fully digital planning procedure were comparable to conventional guided implant surgery. In their study, however, both the global deviations at implant platform and apex were not disclosed, which made it somewhat difficult to justify the comparison of the results.

Regarding the in vivo accuracy of sGIS using conventional planning workflow, a meta-analysis of 14 clinical studies by Zhou et al. [18] reported a mean global deviation of 1.25 mm at the implant platform, 1.57 mm at the implant apex, and a mean angular deviation of 4.1º. Another systematic review of 24 clinical and preclinical studies by Tahmaseb et al. [20] revealed mean total errors of 1.12 mm at the implant platform and 1.39 mm at the implant apex, and a mean angular deviation of 3.89º. The differences in the results of the two studies are reasonable, considering that in vitro studies, which are conducted in a well-controlled environment, normally have a higher accuracy compared to in vivo studies [19]. The present study reported a mean global deviation of 0.78 mm at the implant platform and 1.28 mm at the implant apex; hence, compared with the results of other in vivo studies in the literature, the results of the present study showed greater accuracy in terms of the mean values of positional deviations.

Only tooth-supported surgical guides were used in this study. This may probably be one of the reasons for the better accuracy of this study. In a meta-analysis study, Raico Gallardo et al. [17] concluded that the accuracy was better for tooth-supported guides than bone and mucosa-supported guides. Similar results appear in the literature [20,28]. Nevertheless, the mean lateral deviation at the implant platform and apex (0.57 and 1.14 mm, respectively) of the present study was still smaller than that previously described in the study using a fully digital planning modality by Skjerven et al. [22] (1.05 and 1.63 mm, respectively), in which all implants were also inserted via tooth-supported surgical guides.

However, the mean angular deviation of this study (4.30 ± 2.87º) was higher than the above-mentioned studies with conventional workflow. Skjerven et al. [22] also concluded that the main deviation was angular in their clinical study. Many variables that could affect the accuracy of sGIS have been proposed and investigated, including the location of implant site [29,30,31], jaw position [5,18,32,33], alveolar bone quality [16,30], implant length [31,34], implant diameter [35], guide support [34,36], and the flap or flapless approach [1,4,20,36,37]. In the present study, however, none of the above-mentioned variables were found to have significant influence on the accuracy of sGIS. The only significant difference we observed in the analyzed variables was the registration protocol for matching CBCT and intraoral scans.

In this study, implants planned based on the surface registration protocol showed significantly larger lateral deviation at the implant apex than the fiducial marker registration protocol. This higher lateral deviation at the apex level could be attributed to the effect of angular deviation [30]. In fact, the mean angular deviation for the surface registration protocol (4.96 ± 3.32º) was larger than the fiducial marker registration protocol (3.38 ± 1.81º), with marginal significance (*p* = 0.0526). Studies have shown that the “image-fusion” technique, that is, the superimposition of the CBCT scan and the optical scan of a dental model by dental surface registration, is as accurate as superimposition with the aid of fiducial markers [6,33]. However, the effect of metal artifacts of crown restorations in CBCT image was not considered in these studies.

Schnutenhaus et al. [38] investigated the clinical accuracy of the CBCT matching method. They concluded that matching CBCT data with model scan data was accurate enough for planning sGIS and suggested that the radiographic template could be dispensed with to save time and money of the patient. However, they also emphasized that in cases with many metal restorations, a radiographic template was still needed due to the effect of metallic artifacts of the CBCT. Kim et al. [34] also found a significant impact of metal artifacts within CBCT data on the accuracy of sGIS. Moreover, in the study by Flugge et al. [39], the accuracy of matching the CBCT and intraoral scans by dental surface registration was investigated. They reported that the greater the number of metal restorations in a patient, the less accurate the registration was. In the present study, the surface registration protocol was the first choice to adopt, as long as a radiographic template was not essential to correctly match the CBCT and intraoral scan images. Fifteen patients were planned based on the surface registration protocol. However, all of them had at least one metal crown restoration, and almost half of the patients (seven patients) had more than five metal crowns in the studied jaw. In addition, there were no observed significant differences for any other compared variables of the patient, implant, surgical guide, or surgery; hence, we have reason to believe that the streaking artifact of metal restorations of CBCT can influence the correctness of CBCT/intraoral scan matching, and therefore the accuracy of the GIS. Further investigations are necessary to clarify this issue.

For patients with extensive metallic artifacts in the CBCT image, a fiducial marker protocol was adopted in this study. The patient took the CBCT scan with a radiographic template seated on the dentition. Conventionally, the radiographic template was fabricated manually in a dental laboratory. Moreover, in the so-called “double scan procedure”, the SLA surgical guide was fabricated from the digital data of the radiographic template from the CBCT scan [4]. The three-dimensional reconstruction image of the CBCT data could be influenced by different grey value settings [39]. Surgical guides fabricated from CBCT-scanned casts have been shown to be less accurate than those fabricated from optically scanned casts [40]. Different from the conventional workflow, the fiducial marker protocol in this study used the intraoral scan data to fabricate an SLA radiographic template. This CAD/CAM procedure could eliminate the possible laboratory errors and reduce the inaccuracy from the image segmentation of CBCT data.

To date, the most commonly used method to assess the accuracy of sGIS was the pre- and post-operative CT overlapping [41]. Although commonly applied to the accuracy studies, the major problem of CT matching is that, because of the streaking metal artifacts of the titanium implant, the geometry of the post-operative implant was ambiguous, which could lead to an incorrect estimation of the implant position [3]. Another issue of the CT matching method was that the use of post-operative CBCT might be considered ethically questionable because the patient was exposed to extra radiation dose. A different method using the digital impression technique and implant scan body to identify the implant position has been proposed [3]. This CAD/CAM-based method could reduce the radiation exposure of the patient; it was also reported to be more accurate than the CT matching [42]. The accuracy of these measurement methods should be further investigated.

To be able to compare to other earlier accuracy studies, this study also used the CT matching method to measure the deviations of the placed and planned implants with the consent of the patients and the approval of the ethical committee. However, instead of estimating the implant position manually, we employed a best-fit algorithm of the metrology software to automatically replace the ambiguous post-operative CBCT implant image with an STL file of the implant. This computing processing can minimize the potential errors from manually outlining the implant image; hence, it was helpful to attain a more reliable post-operative implant position.

The major limitation of this fully digital planning workflow is that, currently, it can only be adopted in dentate patients. For the surface registration protocol, more than five remaining teeth were necessary to achieve appropriate matching of the CBCT and intraoral scans [8,38]. In addition, the distribution of the remaining teeth is also important. They should be able to form a triangle as extensive as possible. For the fiducial marker protocol, the major drawback is that extra cost is needed for fabricating the SLA radiographic template. A wrong intraoral seating of the template during a CBCT scan can also lead to error. However, the digital process is more efficient, and errors from conventional laboratory procedures can be reduced. The results of the present study justify the use of the fully digital planning protocol. For partial edentulous patients, the accuracy of sGIS based on this fully digital planning protocol coincides with the conventional workflow.

## 5. Conclusions

Within the limits of the present study, we concluded that:

1. Compared with the results of in vivo studies in the literature, the mean values of the positional deviations of the sGIS planned by a fully digital workflow were smaller than the conventional workflow.

2. A larger mean value of the angular deviation was observed when matching CBCT scan with intraoral scan by the direct registration of the dental surface images, probably owing to the effect of metallic artifact of the crown restorations in CBCT image. Further investigations are necessary to clarify this issue.

3. The clinical accuracy of the sGIS planned by a fully digital workflow was consistent with the conventional workflow. The implementation of the fully digital planning protocol is with satisfactory accuracy so that it may be reliable for partially edentulous patients.

## Figures and Tables

**Table 1:** Deviations between planned and placed implant position by registration protocols.

Variables	Implant Number	Deviations at Implant Platform		Deviations at Implant Apex		Depth Deviation (mm)	Angular Deviation (Degree)
		Global (mm)	Lateral (mm)	Global (mm)	Lateral (mm)		
**Total**	43	0.78 ± 0.39	0.57 ± 0.33	1.28 ± 0.72	1.14 ± 0.72	0.46 ± 0.36	4.30 ± 2.87
(range)		(0.20–1.97)	(0.09–1.52)	(0.18–3.78)	(0.15–3.73)	(0.02–1.49)	(0.73–15.83)
**Registration protocol**							
Dental surface	25	0.81 ± 0.41	0.64 ± 0.36	1.44 ± 0.87	1.34 ± 0.86	0.43 ± 0.33	4.96 ± 3.32
Fiducial marker	18	0.74 ± 0.38	0.47 ± 0.26	1.07 ± 0.34	0.87 ± 0.30	0.50 ± 0.39	3.38 ± 1.81
***p Value***		*0.5620*	*0.1074*	*0.0588*	0.0167 *	*0.5384*	*0.0526*

*p Value*: Student’s t-test, * *p* < 0.05.

**Table 2:** Comparison of deviations for the examined variables.

Variables	Implant Number	Deviations at Implant Platform		Deviations at Implant Apex		Depth Deviation (mm)	Angular Deviation (Degree)
		Global (mm)	Lateral (mm)	Global (mm)	Lateral (mm)		
**Implant site**							
Incisor/canine	9	0.58 ± 0.29	0.53 ± 0.28	1.38 ± 1.08	1.35 ± 1.07	0.20 ± 0.15	4.67 ± 4.42
Premolar	12	0.84 ± 0.35	0.61 ± 0.28	1.32 ± 0.45	1.13 ± 0.52	0.53 ± 0.33	4.00 ± 2.10
Molar	22	0.83 ± 0.44	0.56 ± 0.37	1.23 ± 0.69	1.07 ± 0.65	0.52 ± 0.39	4.32 ± 2.57
***p Value***		*0.2325*	*0.8715*	*0.8506*	*0.6141*	*0.0512*	*0.8750*
**Jaw position**							
Mandible	25	0.85 ± 0.42	0.60 ± 0.36	1.26 ± 0.67	1.10 ± 0.65	0.52 ± 0.38	4.38 ± 2.56
Maxilla	18	0.68 ± 0.33	0.52 ± 0.26	1.32 ± 0.79	1.20 ± 0.82	0.37 ± 0.32	4.20 ± 3.33
***p Value***		*0.1683*	*0.4175*	*0.7685*	*0.6358*	*0.1805*	*0.8481*
**Bone quality**							
Type II	6	0.72 ± 0.30	0.58 ± 0.29	1.18 ± 0.64	1.09 ± 0.64	0.36 ± 0.28	4.05 ± 2.15
Type III	29	0.80 ± 0.38	0.61 ± 0.35	1.37 ± 0.79	1.25 ± 0.80	0.45 ± 0.31	4.66 ± 3.25
Type IV	8	0.75 ± 0.52	0.42 ± 0.23	1.04 ± 0.44	0.78 ± 0.22	0.57 ± 0.55	3.20 ± 1.42
***p Value***		*0.8841*	*0.3385*	*0.4911*	*0.2564*	*0.5358*	*0.4445*
**Implant length**							
8/10 mm	23	0.85 ± 0.43	0.59 ± 0.35	1.27 ± 0.69	1.11 ± 0.65	0.54 ± 0.39	4.62 ± 2.49
11.5 mm	11	0.76 ± 0.35	0.53 ± 0.32	1.20 ± 0.47	1.02 ± 0.55	0.42 ± 0.38	3.48 ± 2.06
13/16 mm	9	0.63 ± 0.31	0.55 ± 0.30	1.41 ± 1.05	1.37 ± 1.05	0.28 ± 0.16	4.49 ± 4.43
***p Value***		*0.3496*	*0.8518*	*0.7990*	*0.5464*	*0.1524*	*0.5552*
**Implant diameter**							
3.5 mm	3	0.52 ± 0.12	0.46 ± 0.20	0.84 ± 0.21	0.81 ± 0.19	0.16 ± 0.14	2.85 ± 1.28
3.75 mm	10	0.73 ± 0.32	0.57 ± 0.32	1.21 ± 0.52	1.11 ± 0.56	0.36 ± 0.30	3.97 ± 1.84
4.2 mm	30	0.82 ± 0.42	0.58 ± 0.34	1.35 ± 0.79	1.19 ± 0.80	0.52 ± 0.37	4.56 ± 3.23
***p Value***		*0.4145*	*0.8514*	*0.4743*	*0.6913*	*0.1544*	*0.5742*
**Guide support**							
Bilaterally	19	0.72 ± 0.37	0.57 ± 0.31	1.39 ± 0.84	1.27 ± 0.86	0.37 ± 0.32	4.22 ± 3.28
Unilaterally	24	0.83 ± 0.41	0.57 ± 0.34	1.21 ± 0.63	1.06 ± 0.62	0.53 ± 0.37	4.36 ± 2.57
***p Value***		*0.3591*	*0.8737*	*0.4388*	*0.3467*	*0.1326*	*0.6510*
**Technique**							
Open flap	16	0.80 ± 0.38	0.59 ± 0.25	1.41 ± 0.84	1.27 ± 0.82	0.46 ± 0.41	4.75 ± 3.82
Flapless	27	0.77 ± 0.41	0.55 ± 0.37	1.21 ± 0.64	1.06 ± 0.65	0.45 ± 0.33	4.03 ± 2.17
***p Value***		*0.7900*	*0.7159*	*0.3979*	*0.3580*	*0.9517*	*0.4970*

*p Value*: Student’s t-test or ANOVA test.
